# Supplementary material for: Zebrafish preserve global germline DNA methylation while sex-linked rDNA is amplified and demethylated during feminisation
Source: Nat Commun. 2019 Jul 16;10:3053. doi: 10.1038/s41467-019-10894-7 (PMC6635516; doi:10.1038/s41467-019-10894-7)
Supplement: Supplementary file 1 — Supplementary Information [file 41467_2019_10894_MOESM1_ESM.pdf]

Supplementary information

Zebrafish preserve global germline DNA methylation while sex-linked rDNA is amplified and demethylated during feminisation, Ortega-Recalde *et al.*

Supplementary Figures

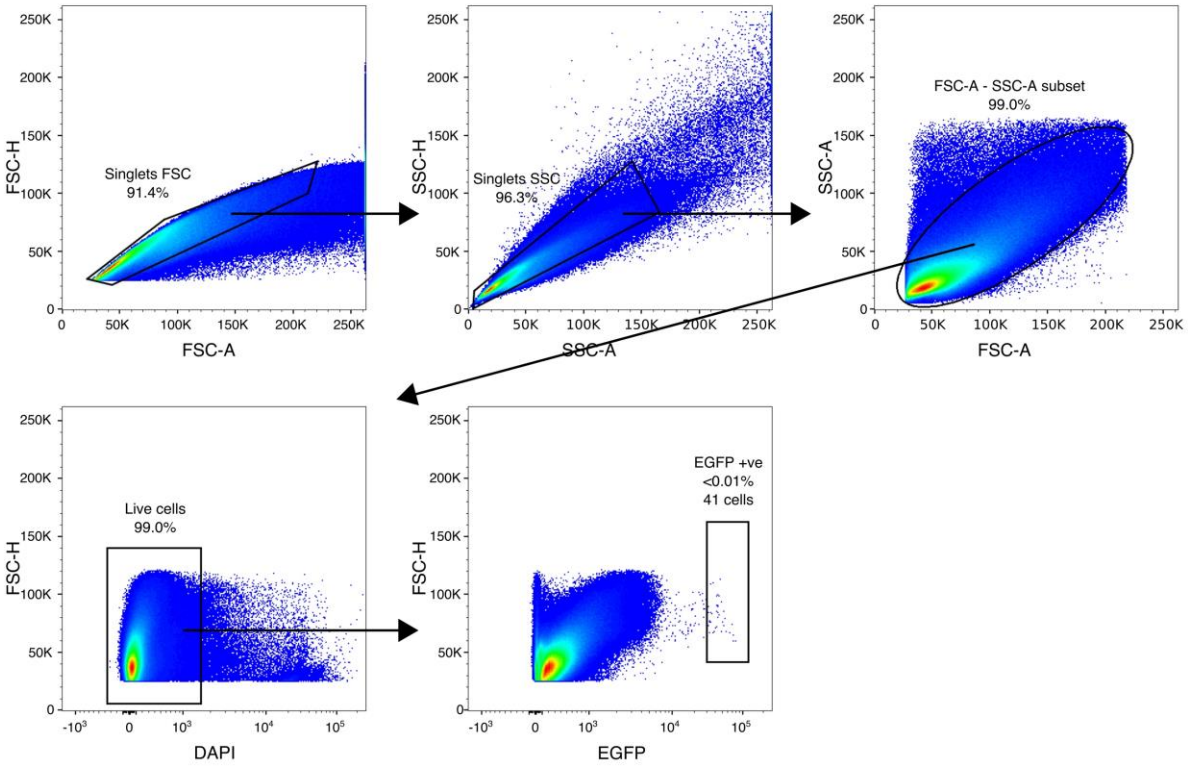

Supplementary Figure 1. *Gating strategy used for cell sorting.* Cells were first gated by forward and side scatters (FSC and SSC respectively) to exclude doublets. The live cell gate was then set to eliminate dead cells stained with DAPI. The gate boundaries for ‘positive’ and ‘negative’ cells were determined within the live cell gate by three criteria: i.) large EGFP intensity differences. ii.) cell clustering. iii.) EGFP expression in tissues containing non-germline cells. The same strategy was used to sort vasa:EGFP +ve cells for results presented on Fig. 1B, 1C, 2G and 2H. Blue dots indicate discrete data points (i.e. cellular events), whereas green, yellow and red colouring indicate increasing data density.

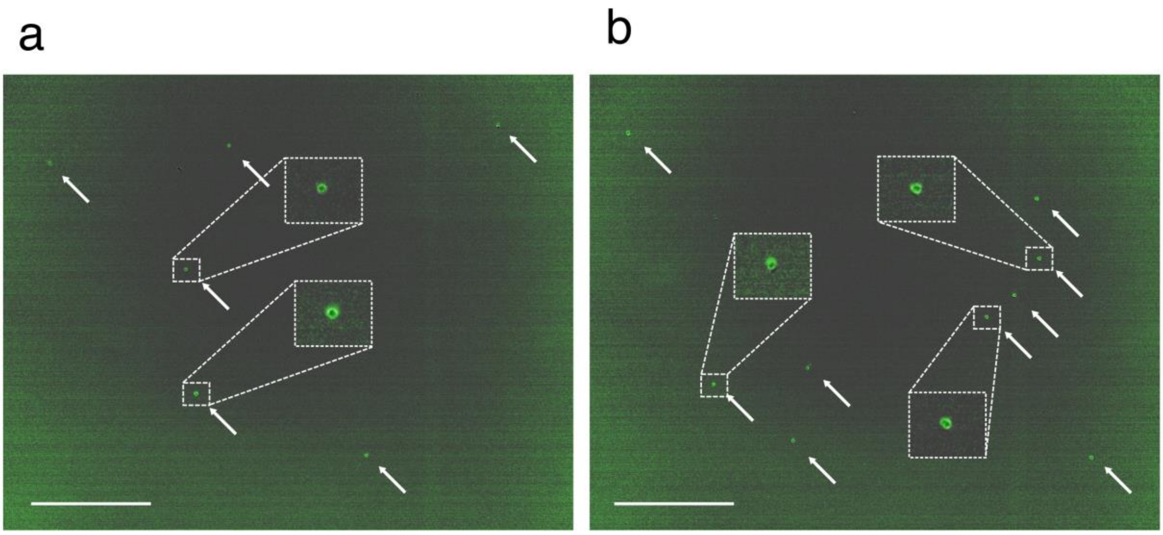

Supplementary Figure 2. *Germline isolation by FACS.* **a-b** Fluorescence microscopy of vasa:EGFP +ve sorted cells. Magnification of EGFP +ve labelled cells is shown inset (dashed lines). The proportion of EGFP +ve cells ranged from 93.8% to 100%. Scale bars are 400 µm.

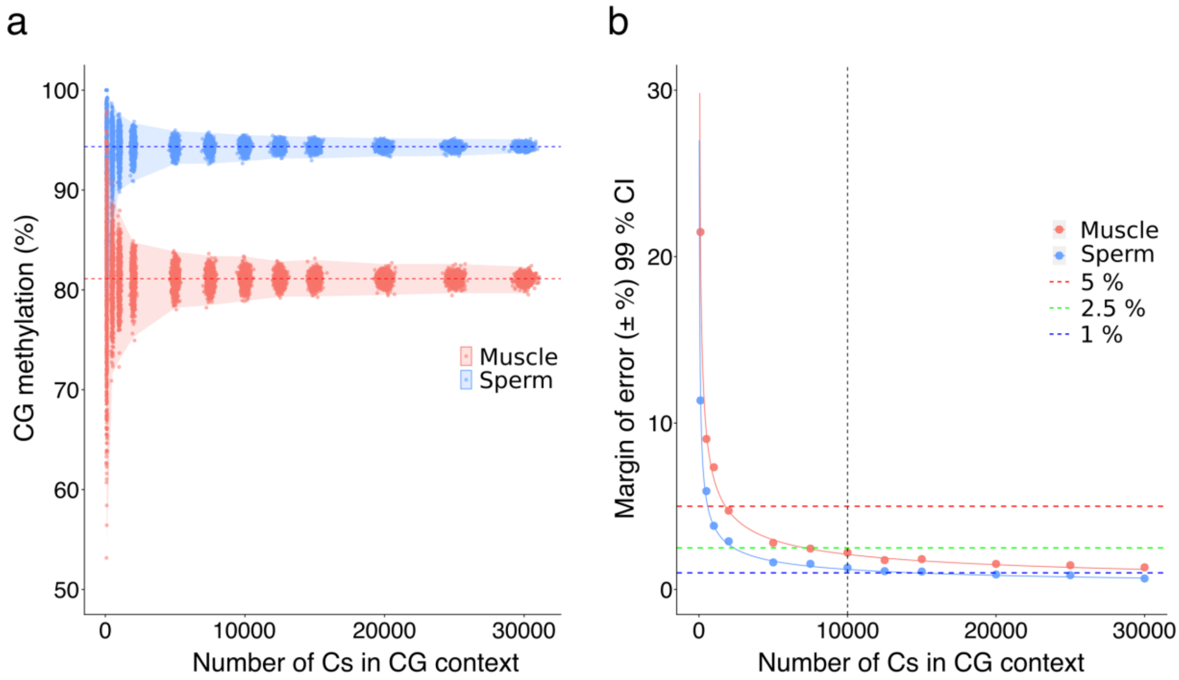

Supplementary Figure 3. *Empirical bootstrap sampling of high coverage zebrafish methylation datasets.* **a** Percentage of methylation per number of Cs sampled for 1,000 replicates at each sample size. **b** Confidence Interval (CI) at different sample sizes. Margin of error ± 99% CI (*n* = 1,000).

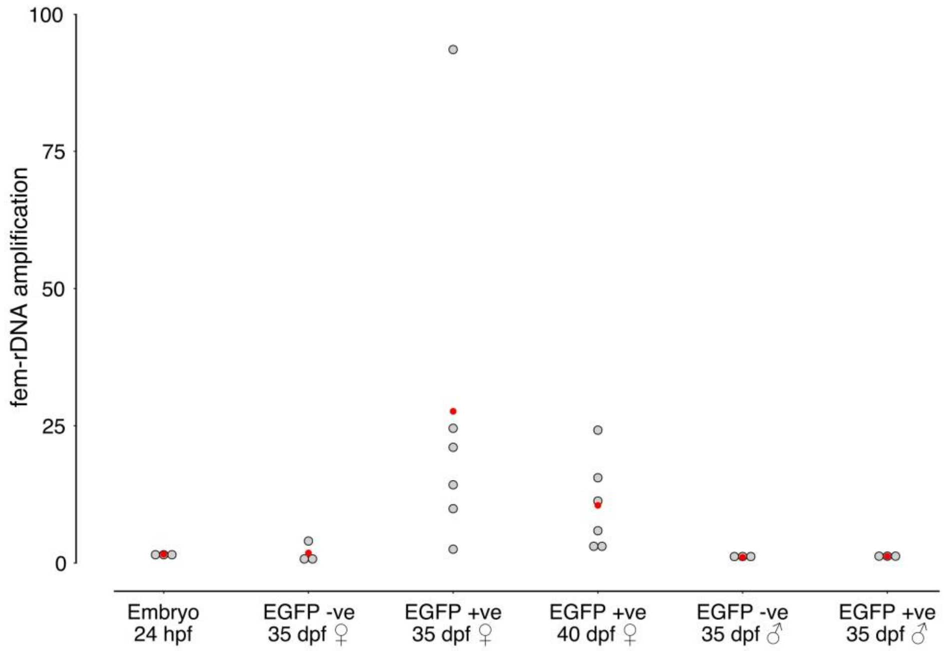

Supplementary Figure 4. *Amplification of fem-rDNA determined by quantitative PCR.* A baseline level of amplification was determined as the mean of Δ Ct (Ct fem-rDNA – Ct som-rDNA) for non-germline samples (EGFP -ve females and males) and the value obtained was used as control for the ΔΔ Ct method (n = 3 non-germline samples, EGFP +ve males and embryos at 24 hpf, n = 6 EGFP +ve 35 and 40 dpf females). Arithmetic means are represented by red dots.

Supplementary Table

Supplementary Table 1. *List of oligonucleotides used in this study.*

**Name**	**Sequence**
BioP5N7	[Bio] - ACACTCTTTCCCTACACGACGCTCTTCCGATCTNNNNNNN
P7N7	GTGACTGGAGTTCAGACGTGTGCTCTTCCGATCTNNNNNNN
Zf_fem_rDNA_1F	ACACACATTGATCATCGACCT
Zf_fem_rDNA_1R	TAGCAAGGCGACCCTCAGA
Zf_som_rDNA_1F	GATGCCCTTAGCTGGGTGT
Zf_som_rDNA_1R	CCTAGCTGCGGTATTCAGCG
