# Supplementary material for: Zebrafish preserve global germline DNA methylation while sex-linked rDNA is amplified and demethylated during feminisation
Source: Nat Commun. 2019 Jul 16;10:3053. doi: 10.1038/s41467-019-10894-7 (PMC6635516; doi:10.1038/s41467-019-10894-7)
Supplement: Supplementary file 3 — Description of Additional Supplementary Files [file 41467_2019_10894_MOESM3_ESM.pdf]

Description of Additional Supplementary Files

File name: Supplementary Data 1

Description: *Low coverage bisulfite sequencing of zebrafish germinal cells and control somatic tissues.* The table lists the general sequencing statistics as well as the number of cytosine calls at either CG dinucleotides ('CG') or in other sequence contexts ('non-CG'), for germline cells or control cells, mapped against the Zebrafish genome assembly version 11 (GRCz11). Details of bioinformatic processing are provided in the Methods section. The frequency of non-CG methylation indicates the maximum rate of non-conversion during the bisulfite treatment step; by this measure, all libraries had a bisulfite conversion efficiency of at least 96.02%.

File name: Supplementary Data 2

Description: *Conversion sex-linked SNPs Zv9 to GRCz11.* Conversion of genome coordinates from published zebrafish sex-linked SNPs (Wilson *et al*, 2014) between Zv9 and GRCz11. Only results located on chromosome 4 are shown.

File name: Supplementary Data 3

Description: *Amplification and methylation of fem-rDNA.* Amplification and methylation of fem-rDNA. Total number of reads mapping to GRCz11 is indicated in column E (Total unique mapped reads), as is the number mapping to fem-rDNA, column F(chr4:77,549,891-77,567,278). CG calls originating from those reads are shown in columns I-J.

File name: Supplementary Data 4

Description: *fem-rDNA amplification and demethylation at different developmental stages.* Number of reads, CG calls and percentage of metylation for fem-rDNA at different developmental stages (Potok *et al*. 2013)
